# A multi‐organ analysis of the role of mTOR in fetal alcohol spectrum disorders

**DOI:** 10.1096/fj.202201865R

**Published:** 2023-03-31

**Authors:** Alexander L. Carabulea, Joseph D. Janeski, Vishal D. Naik, Kang Chen, Gil Mor, Jayanth Ramadoss

**Affiliations:** ^1^ Department of Obstetrics & Gynecology, C.S. Mott Center for Human growth and Development, School of Medicine Wayne State University Detroit Michigan USA; ^2^ Barbara Ann Karmanos Cancer Institute Wayne State University Detroit Michigan USA; ^3^ Department of Physiology, School of Medicine Wayne State University Detroit Michigan USA

**Keywords:** alcohol, autophagy, development, fetal, mTOR, pregnancy

## Abstract

Alcohol exposure during gestation can lead to fetal alcohol spectrum disorders (FASD), an array of cognitive and physical developmental impairments. Over the past two and a half decades, Mammalian Target of Rapamycin (mTOR) has emerged at the nexus of many fields of study, and has recently been implicated in FASD etiology. mTOR plays an integral role in modulating anabolic and catabolic activities, including protein synthesis and autophagy. These processes are vital for proper development and can have long lasting effects following alcohol exposure, such as impaired hippocampal and synapse formation, reduced brain size, as well as cognitive, behavioral, and memory impairments. We highlight recent advances in the field of FASD, primarily with regard to animal model discoveries and discuss the interaction between alcohol and mTOR in the context of various tissues, including brain, placenta, bone, and muscle, with respect to developmental alcohol exposure paradigms. The current review focuses on novel FASD research within the context of the mTOR signaling and sheds light on mechanistic etiologies at various biological levels including molecular, cellular, and functional, across multiple stages of development and illuminates the dichotomy between the different mTOR complexes and their unique signaling roles.

## Graphical abstract

Interaction between alcohol and mTOR complexes and their unique signaling roles in brain, placenta, muscle, and bone with respect to developmental alcohol exposure at various biological levels across multiple stages of development.

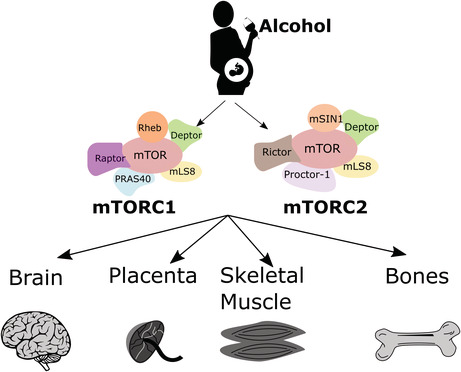

## 1 INTRODUCTION

Fetal alcohol spectrum disorders (FASD) is an overarching term that encompasses a range of developmental outcomes exhibited in children exposed to alcohol in utero.^1^ FASD can present differently in every child, however, intellectual and/or behavioral impairments are always reported. The most severe of these disorders is Fetal Alcohol Syndrome (FAS), which is characterized by facial dysmorphology, growth restriction, and central nervous system/neurodevelopmental abnormalities.^2^ As a result, the US Surgeon General and the American Academy of Pediatrics have issued an advisory to abstain from any alcohol when considering pregnancy and throughout pregnancy.^3^, ^4^ Although the exact mechanism of FASDs remains elusive, the Mechanistic Target of Rapamycin (mTOR) has emerged as a key control node in a network that responds to metabolic signals and has been implicated in various tissue abnormalities following alcohol pathology.

mTOR is one of six phosphatidylinositol 3‐kinase (PI3K)‐related kinase (PIKK) family members, which are implicated in a variety of cellular functions anywhere from DNA damage sensing and repair to cell cycle progression and arrest.^5^, ^6^, ^7^ More specifically, mTOR is a 289‐kDa serine/threonine protein kinase that makes up the catalytic subunit of two individual complexes, known as mTOR Complex 1 (mTORC1) and mTOR Complex 2 (mTORC2). These complexes each have distinctive substrates and functions, in addition to unique accessory proteins and sensitivity to rapamycin.^6^, ^8^, ^9^ It is important to note that rapamycin directly inhibits mTORC1, while it only indirectly inhibits the rapamycin‐insensitive counterpart in mTORC2.^10^

mTORC1 plays a pivotal role in bringing multiple cellular signals together to gauge the availability of nutrients, growth factors, and energy to promote cellular growth and catabolic activity during stress. mTORC1 maintains tight regulation over translation, as this is among the most energy‐ and resource‐intensive cellular processes.^6^ It is also critical that cellular building blocks are not broken down again before they can be incorporated into their respective processes, therefore it is crucial that mTOR regulates and suppresses catabolic autophagy.^11^ By blocking autophagy, cells are allowed to accumulate proteins and organelles, even if they are redundant or damaged. Not much is known about mTORC2 and its relation to these processes, however recent studies have shown that mTORC2 may promote mTORC1 activity.^6^, ^12^

To our knowledge, this is the first review that examines the effects of gestational alcohol exposure on the mTOR signaling pathway. In this review, we analyze studies that examine mTOR, alcohol, and their relation to pregnancy. Our review presents a detailed analysis of various tissues implicated in FASD and potential areas of study including brain, placenta, bone, and skeletal muscle.

## 2 mTOR ARCHITECHTURE AND SIGNALING PATHWAY

### 2.1 mTOR architechture

mTORC1 is made up of three core components: mTOR, Target of rapamycin complex subunit LST8 (mLST8), and its defining scaffold protein Regulatory‐Associated Protein of mTOR (RAPTOR).^6^ This RAPTOR scaffold facilitates the mTORC1 accessory factor, Proline‐Rich AKT Substrate 40 kDa (PRAS40), which behaves as an mTORC1 inhibitor alongside DEP‐domain‐containing mTOR‐interacting protein (DEPTOR). RAPTOR is also necessary for intracellular localization of mTORC1 and plays a role in recruiting substrates by binding to respective TOR signaling motifs. While the core of mTORC2 also includes mTOR and mLST8, it is instead defined by the scaffold protein Rapamycin‐Insensitive Companion of mTOR (RICTOR), which binds to Mammalian Stress‐activated MAP Kinase‐interacting Protein 1(mSIN1), DEPTOR, and protein associated with rictor 1 or 2 (PROTOR1/2).^6^

### 2.2 mTORC1 signaling pathway and protein synthesis

**FIGURE 1:**
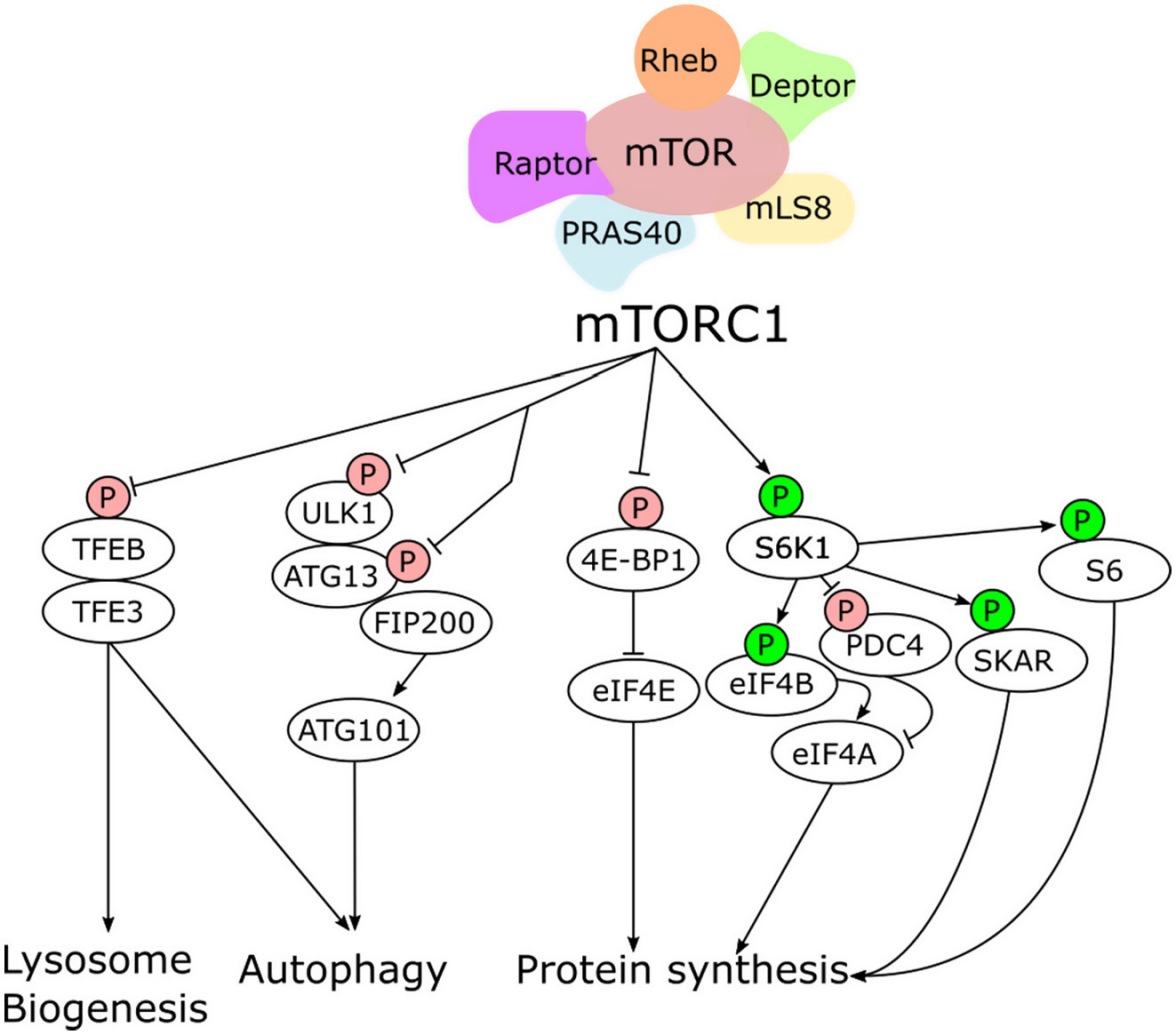
Downstream targets of mTORC1 signaling. Activation of mTORC1 initiates a downstream cascade that enhances the production of proteins, lipids, nucleotides, and other macromolecules. In tandem with this anabolic paradigm, mTORC1 inhibits catabolic processes, such as autophagy and lysosome biogenesis.

To increase the production of proteins, lipids, nucleotides, and ATP, while maintaining a balance with catabolic activity, mTORC1 must phosphorylate the appropriate substrates (Figure 1). mTORC1 directly promotes protein synthesis by ultimately phosphorylating the Eukaryotic Translation Initiation Factor 4E‐binding Proteins (4E‐BPs), as well as Ribosomal Protein S6 Kinase 1 (S6K1). Along with Phosphoinositide‐dependent Kinase (PDK), which phosphorylates the activation loop (Thre229), mTORC1 phosphorylates S6K1 on its hydrophobic motif (Thre389), which stimulates its kinase activity.^13^, ^14^ mTORC1 and S6K1 in turn amplify RNA Polymerase I and RNA Polymerase III activity via phosphorylation of the Upstream Binding Factor (UBF)^15^ regulatory factors, Transcription Initiation Factor IA (TIF‐IA),^16^ and RNA polymerase III transcription MAF1 homolog (MAF1),^17^, ^18^ which results in the upregulated rRNA transcription. S6K1 can also enhance translation via activation of Eukaryotic Translation Initiation Factor 4B (eIF4B),^19^ as well as by degrading the Eukaryotic Translation Initiation Factor 4A (eIF4A), inhibitor programmed cell death 4 (PDCD4),^20^ and by associating with Polymerase Delta‐Interacting Protein 3 (POLDIP3)^21^ at exon junction complexes.

### 2.3 mTORC1 signaling pathway and autophagy

Normally mTORC1 phosphorylates, and in turn inhibits Unc‐51‐like Autophagy‐activating Kinase 1 (ULK1)^22^ at Ser757 and Autophagy‐related 13 (ATG13)^23^ at Ser355. These important effectors of autophagy induction, in tandem with 200‐kDA FAK family kinase‐interacting protein (FIP200) and Autophagy Related 101 (ATG101),^24^, ^25^ spur formation of the autophagosome. However, during nutrient deprivation, inhibition of mTORC1 allows these molecules to resume autophagosome initiation, as well as permit the nuclear translocation of the Transcription Factor EB (TFEB)^26^ and the Transcription Factor E3 (TFE3)^27^ to coordinate lysosomal biogenesis. mTORC1 facilitates both protein synthesis and autophagy via the aforementioned signaling pathways, with little overlap aside from environmental cues. Herein we will review alcohol's effect on mTOR machinery in various tissues within the context of binge alcohol exposure, fetal development, and elucidate future directions.

## 3 INTERACTIONS OF ALCOHOL AND mTOR ON BRAIN DEVELOPMENT

The mTOR signaling pathway is closely associated with normal fetal and postnatal brain development and is also necessary for growth, survival, and differentiation of neural stem cells.^10^ Normal postnatal development of brain size is regulated by mTOR signaling and mice lacking mTOR exhibit smaller brain size with fewer neurons and neural progenitors.^28^ Exposure to alcohol during development alters expression of proteins involved in both neuronal growth and differentiation via the mTOR signaling pathway, and can lead to oxidative stress.^29^ Herein, we will discuss how mTOR signaling, translation dysregulation, and neuroprotection mediated by autophagy can occur in multiple brain regions such as the cortex, hippocampus, and cerebellum following prenatal alcohol exposure.

### 3.1 Brain mTORC1 dysregulation in FASD

Alcohol interacts with mTOR to impair protein synthesis in multiple brain regions. In a study, cortical neurons were isolated from Postnatal Day (PND) 14 C57BL/6 mouse fetuses and cultured in 100 mM alcohol for 5 days.^29^ These neurons were then used in a microarray consisting of 638 sequence‐verified genes to identify pathways significantly modulated following alcohol exposure. Notably, mRNA expression of FKBP rapamycin‐associated protein was decreased by 22%, which associates with mTOR to restrict access of mTOR substrates to the complex.^29^ Eukaryotic Translation Initiation Factor 4E (eIF4E), which is an mRNA 5′‐cap binding protein, was also decreased by 24%, and mitogen‐activated protein kinase kinase (MAPKK) decreased by 24% as well, which phosphorylates eIF4E. These results suggest alcohol exposure represses the kinase activity of mTOR and leads to decreased activation of the downstream substrate Eukaryotic Translation Initiation Factor 4E‐Binding Protein 1 (4E‐BP1). Hypophosphorylated 4E‐BP1 in turn competes with Eukaryotic Translation Initiation factor 4G (eIF4G) for a binding site on eIF4E and the resultant eIF4E/4E‐BP complexes impair translation by inhibiting binding of eIF4E to the mRNA cap structure, thereby hindering protein synthesis and possibly resulting in apoptosis.^29^

**FIGURE 2:**
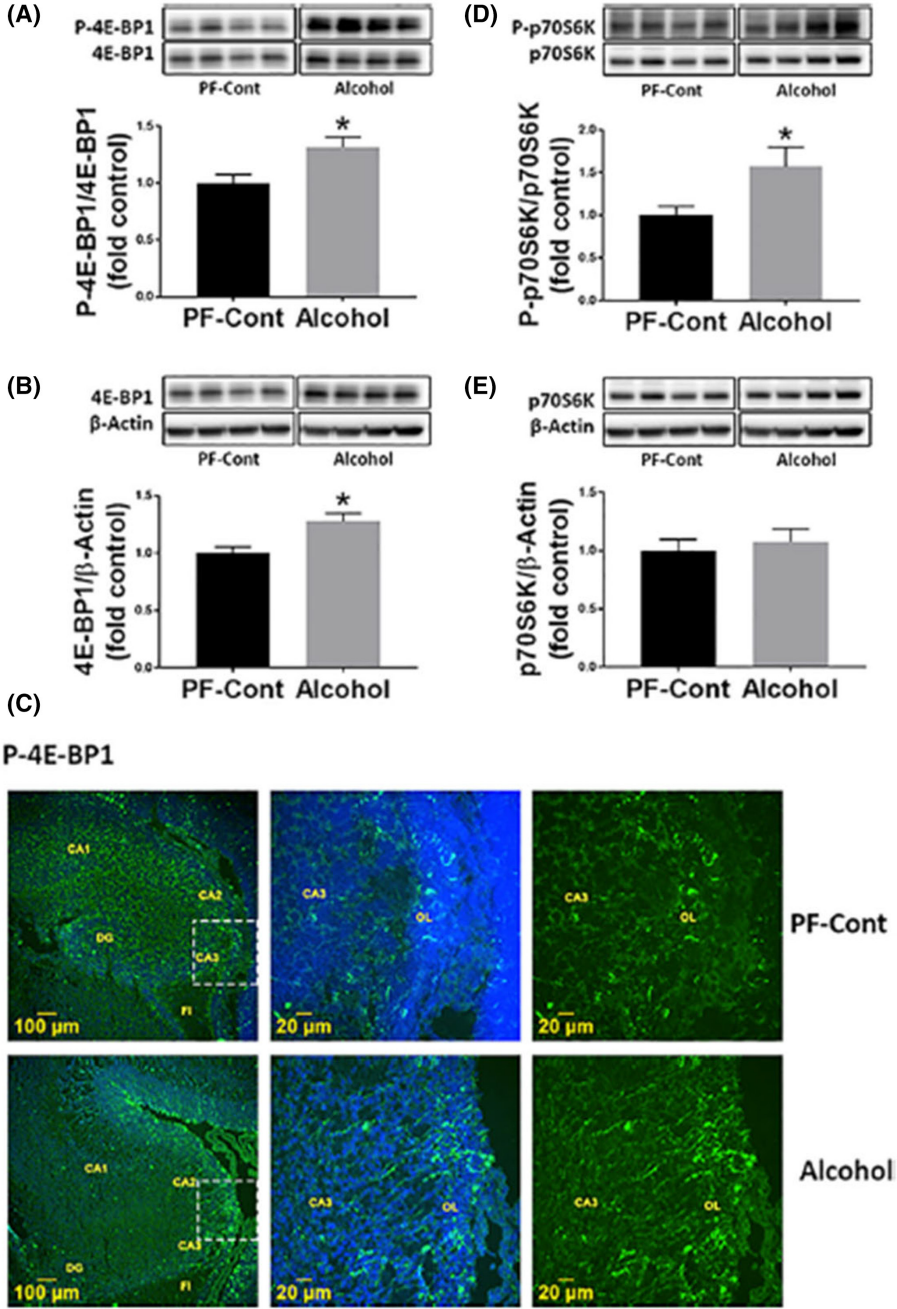
Effect of gestational chronic binge alcohol exposure on mTORC1 signaling in the fetal hippocampus. Immunoblot analysis showed that gestational chronic binge alcohol exposure significantly increased (A) P‐4E‐BP1 level (↑ 41%; *p* = .0156) and (B) total 4E‐BP1 expression (↑ 20%; *p* = .0251) in the fetal hippocampi. (C) Immunofluorescence staining shows that P‐4E‐BP1 was expressed in the oriens layer (OL) of the fetal hippocampus and the representative image shows CA3 field near fimbria (FI). (D) Gestational alcohol exposure significantly increased (↑ 57%; *p* = .0383) the level of P‐p70 S6K, whereas (E) total p70 S6K expression was not different between groups. Data are shown as mean ± SEM and as fold of control. *Significance was established a priori at *p* < .05. Adapted with permission from Lee et al.^31^ (PF‐Cont = Pair‐fed Control).

We have previously reported there was a strong effect observed in the hippocampal proteome of Sprague–Dawley rats given 6 g/kg alcohol from Gestational Day (GD) 11–20 following a short 4.5 g/kg acclimation period (GD 5–10). Of note, this was the most vulnerable brain region studied in terms of protein changes, with over 600 significantly altered proteins.^30^ Many of these dysregulated proteins are regulators of cellular growth and developmental morphogenesis, and are specifically involved in the mTOR signaling pathway. Another study with a similar alcohol exposure paradigm (4.5 g/kg GD 5–10, 6 g/kg GD 11–20) in Sprague–Dawley rats showed that alcohol also dysregulates mTORC1 signaling and alters proteins that complex with mTOR in the fetal hippocampus.^31^ Of note, there is a decrease in p‐mTOR (Ser2448) and an increase in DEPTOR expression levels in the fetal hippocampus, which can inhibit mTORC1 signaling following alcohol exposure. An increase in total 4E‐BP1 expression also infers a dysregulation of mTORC1 signaling, along with an increase in phosphorylation levels of 4E‐BP1 and S6K1 (Figure 2), which are phosphorylated downstream of mTORC1 to initiate protein synthesis.^31^

Exposure to alcohol over PND4‐9, a period in rodent brain development comparable to the third trimester in humans, impairs hippocampal neurodevelopment and reduces normal dendritic spine density, neurogenesis, and long‐term potentiation. A study has demonstrated that taurine and choline levels are decreased, whereas glutamate and glutamine are increased in the dentate gyrus of rats exposed to alcohol between PND4‐9 (5 g/kg/day, intragastrically).^32^ However, pretreatment with rapamycin directly before the aforementioned alcohol administration (3 and 10 mg/kg, intraperitoneally) protected dentate gyrus cells against changes in concentration of taurine, choline, glutamate, and glutamine induced by neonatal alcohol exposure. These nutrients are crucial for proper neurodevelopment and these results implicate an interaction between alcohol and mTOR in regulation of amino acid concentrations. Of note, adult male rats (PND60) also had the deleterious effects following neonatal alcohol exposure on the dentate gyrus prevented when administered a rapamycin pre‐treatment.^32^ These discoveries show that exposure to alcohol during development not only disrupts the mTOR signaling pathway, but that these nutrients can in fact be rescued following rapamycin pre‐treatment.

### 3.2 Brain mTORC2 signaling disruptions in FASD

Few studies have been done to elucidate mTORC2 and its role in FASD pathology, however sufficient ground work has been laid to warrant further exploration of the mTORC1 and mTORC2 dichotomy. For example, a deep‐sequencing analysis of fetal cortexes obtained from C57BL/6J mice exposed to alcohol (2.9 g/kg, GD7‐17intraperitoneal injections) revealed 105 out of 109 identified differentially expressed genes were targets of mTORC2.^33^ The authors concluded that the dysregulation and subsequent suppression of genetic pathways regulating mitochondrial dysfunction, oxidative phosphorylation, and EIF2 Signaling is unique to the cortex of alcohol‐exposed brains and are regulated specifically by mTORC2. Chang et al. also observed that multiple members of the mTORC2 complex were upregulated in the alcohol‐exposed fetal cortexes, in addition to downregulation of mTORC2 targets which are normally negatively regulated by mTORC2. Fetal cerebral cortical neuroepithelial stem cells derived from C57BL/6 mouse fetuses (GD12.5) cultured in alcohol containing medium (0, 160, and 240 mg/dL) also reproduced the suppression of mTORC2 target genes *Atp5e* and *Atp5mf*.^33^ These genes are notable as they directly impact mitochondrial function and metabolism. In addition to these discoveries, a study by Lee et al. revealed increased expression of RICTOR in the fetal hippocampus following binge‐alcohol exposure in Sprague–Dawley dams (4.5–6 g/kg, GD5‐21).^31^ mTORC2 has a documented role in establishing epigenetic changes in regard to gene functions and can lead to alterations in both metabolism and oxidative respiration following alcohol exposure,^33^ however more work needs to be done to elucidate the cooperation between mTORC1 and mMTORC2 with regard to oxidative stress responses.

### 3.3 Changes in brain autophagic flux attributed to mTOR signaling disruptions in FASD

Alcohol and mTOR interact to regulate autophagy. The process of autophagy, also known as autophagic flux, includes the sequential steps of phagophore nucleation or initiation, elongation and subsequent maturation of cargo sequestration, as well as degradation of the sequestered cargo.^25^, ^34^ This tightly regulated pathway is controlled by a group of autophagy‐related genes (ATG) and leads to lysosomal degradation and turnover of cytoplasmic organelles and proteins. As a neuroteratogen, exposure to alcohol produces reactive oxygen species (ROS) that can result in oxidative stress in the developing brain. This oxidative stress can cause neuronal death mainly in the form of apoptosis, which has been known to be modulated by autophagy.^34^

**FIGURE 3:**
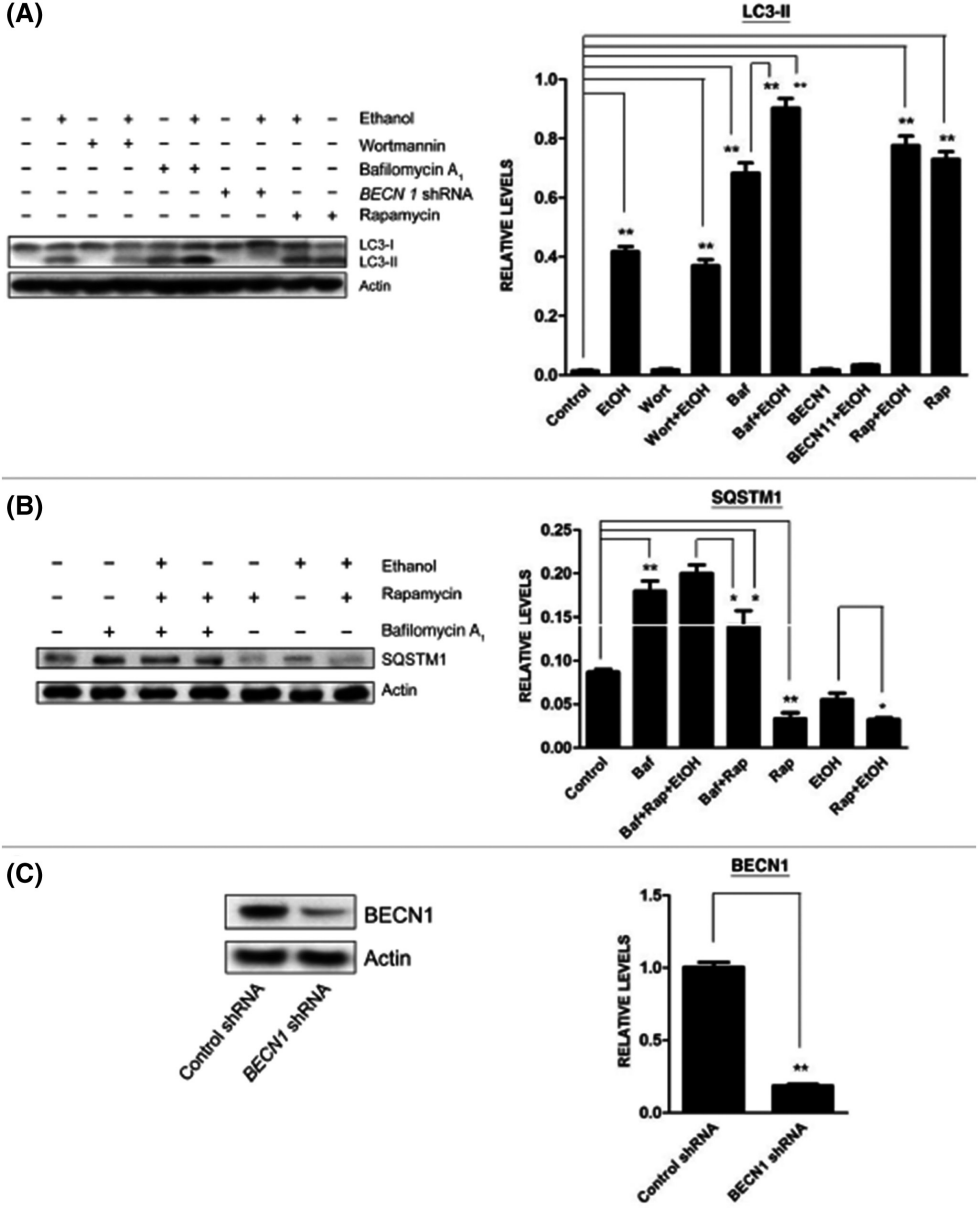
Effect of ethanol on autophagic flux in SH‐SY5Y cells. SH‐SY5Y cells were treated with ethanol (0% or 0.8%) in the presence/absence of wortmannin (Wort: 10 μM), Bafilomycin A1 (Baf: 10 nM), rapamycin (Rap: 10 nM) or *BECN1* shRNA (BEC N1). The protein samples were collected 8 h after the treatment. The levels of LC3 (A) and SQSTM1 (B) were examined with immunoblotting (top panel). The experiment was replicated three times. Relative levels of LC3‐II or SQSTM1 were determined by densitometry and normalized to Actin levels (bottom panel). *p < .05, **p < .01. (C) Cells were transfected with *BECN1* shRNA for 24 h and downregulation of BEC N1 by shRNA was confirmed by immunoblotting (top panel). Relative BEC N1 level was determined by densitometry and normalized to Actin levels (bottom panel). **p < .01. Adapted from Chen et al.^34^ open access.

SH‐SY5Y neuroblastoma cells treated with 0.4% alcohol in medium and PND7 mice injected twice subcutaneously with alcohol (2.5 g/kg) over 8 h showed that alcohol‐induced LC3 lipidation, a marker for autophagy,^35^ was mediated by increasing autophagic flux rather than by inhibiting autophagosome degradation.^34^ This suggests that alcohol increased autophagic activity rather than block the fusion of autophagosomes with lysosomes during development. mTOR is a known regulator of autophagy and may be an important pathway in the induction of a cellular self‐protective response to alleviate alcohol‐induced oxidative stress in the fetal mouse brain. For example, rapamycin, an inhibitor of mTOR and an initiator of autophagy, has been shown to further increase alcohol‐induced upregulation of LC3 lipidation, as well as the formation of autophagosomes (Figure 3).^34^ Notably, Chen et al. went on to demonstrate that stimulation of autophagic flux by rapamycin decreased alcohol‐induced ROS production and also confirmed that antioxidants alone alleviated alcohol‐stimulated autophagy. Therefore, mTOR signaling modulation may activate autophagy as a result of alcohol neurotoxicity and is important for maintaining cellular homeostasis in pregnancy.^30^

### 3.4 mTOR & neurodevelopment dysregulation following alcohol exposure

mTOR not only regulates autophagic machinery as a means to reduce the burden of oxidative stress brought on by alcohol, but mTOR is also a crucial signaling pathway for dendritic pruning. Dendritic pruning is an essential part of normal brain development, selectively removing inappropriate connections that form during the early stages of neuronal growth.^36^ A binge alcohol paradigm, in which adolescent PND30‐43 C57BL/6 mice were given daily intraperitoneal injections (3 mg/kg), reported a greater number of dendritic spines in dentate gyrus and granule cells, suggestive of reduced pruning in the hippocampus.^36^ Additional 3D analysis of spines performed using z‐stacks, noted an increase in thin spines in females and stubby spines in males. This result was accompanied by lower levels of LC3‐II, implicating autophagy is responsible for dendritic pruning.^36^ The addition of rapamycin returned the number of dendritic spines to a normal density, as well as restored normal morphology, resulting in hippocampal dendritic spines similar to those in normal development. This shows that alcohol induces changes in mTOR signaling, which in turn mediates autophagic activity, and can result in improper dendritic pruning and neurodevelopment.

Another study cultured cortical microvessels on PND2 from NMRI mice in medium with 50 mM alcohol in the presence or absence of 200 nM rapamycin.^37^ Girault et al. confirmed a reduction in mTOR signaling along with an upregulation of LC3 lipidation following alcohol treatment in mouse brain endothelial cells. They also showed that autophagy is responsible for protection in both astrocytes and neurons, and suggest that this neuroprotection provided by autophagy may result from mitophagy, or specifically the removal of damaged mitochondria. Notably, the reduction in mTOR signaling was correlated with a reduction in neuronal length, which indicates altered vascular plasticity, as rapamycin prevented this effect.^37^ This work demonstrates that proper mTOR signaling is likely important for maintaining autophagy, vascular plasticity and therefore proper neurodevelopment.

### 3.5 mTOR linked to cognition, behavior, and memory impairments following alcohol exposure

In addition to the interaction between mTOR and autophagy as an important driver of ROS clearance, synaptic pruning, and appropriate vascular plasticity following alcohol exposure in neurodevelopment, autophagy is implicated in the maintenance of long‐term cognition and memory function as well. During memory formation, mTOR is activated in multiple regions of the brain, however a critical control step in synaptic plasticity and memory formation is the regulation of hippocampal protein synthesis.^38^, ^39^ In this context, behavioral tests including Hebb‐Williams mazes, novel object recognition, and passive avoidance tests were conducted in C57BL/6 mice exposed to alcohol from PND30‐43 (3 g/kg).^36^ Interestingly, these mice required significantly more time to complete each maze, and in fact, failed to recognize novel objects, displayed a significantly shorter discrimination index on the object recognition task, and also a shorter latency during passive avoidance tests compared with those in both saline‐treated and rapamycin‐treated groups. Rapamycin restored low cAMP‐responsive element‐binding protein (CREB) levels, a cellular transcription factor implicated in neuronal plasticity and long‐term memory formation in the hippocampus, following alcohol exposure.^36^ Other studies done in alcohol‐exposed PND4‐9 Wistar rats (5 g/kg) confirmed cognitive impairments, spatial learning impairments, and long‐term recognition memory impairments, and showed that pre‐treatment with rapamycin (3 and 10 mg/kg) ameliorated these effects.^32^, ^40^ Lopatynska‐Mazurek et al. attributed these cognitive impairments to overall challenges in spatial processing and changes in biomarkers within the DG,^32^ all of which were reversed in rapamycin‐treated rats. Taken together, these results suggest alcohol can lead to cognitive alterations and poor retention in memory tasks, and supports the contention that mTOR regulates synaptic plasticity, memory storage, and cognition.

### 3.6 Activation of autophagic machinery via mTOR

Let us examine multiple ways alcohol has been demonstrated to interact with mTOR to produce a neuroprotective response. At the molecular level, mTORC1 regulates autophagy through modulation of specific proteins. For example, normally mTORC1 phosphorylates ATG13 and ULK1/2, which in turn inhibits the activity of the ATG13‐ULK1/2‐RB1CC1/FIP200‐ATG101 complex and prevents the activation of autophagy.^34^ However, alcohol can inhibit phosphorylation of two main mTORC1 substrates: 4E‐BP1 and S6K1. It was also reported that rapamycin and alcohol together produced greater dephosphorylation of S6K1 and 4E‐BP1 than did treatment of cells with either rapamycin or alcohol alone. Both rapamycin and a knockdown of mTOR by siRNA each offered neurons protection against alcohol‐induced apoptosis and show the importance of the mTORC1 pathway in alcohol‐induced autophagy and neurotoxicity.^41^

Another potential mechanism for activation of autophagy following alcohol exposure is via adenosine 5′‐monophosphate‐activated protein kinase (AMPK), which is upstream of mTORC1 and also plays a critical role in regulating autophagy in neurons.^41^ Normally, the activation of AMPK inhibits mTORC1 activity through the phosphorylation of the mTORC1‐associated proteins RAPTOR and Tuberous Sclerosis 2 (TSC2), thereby activating autophagy. AMPK is activated via oxidative stress, which is a requirement for ROS‐stimulated autophagy, and indicates that alcohol can activate autophagy by regulating AMPK in neuronal cells.^41^

These results support the role of mTOR‐regulated autophagy in oxidative stress relief, alterations in vascular plasticity, synaptic spine changes, along with memory and cognitive dysfunction in multiple brain regions induced by binge‐like exposure to alcohol. It was also shown that inhibition of mTOR via rapamycin or knockdown of mTOR offers neuroprotection against alcohol‐induced cell death, further supporting that the mTOR pathway is involved in alcohol‐induced autophagy and cell death.^41^ Therefore, it is likely that alcohol modulates autophagy in the brain by inhibiting the mTOR pathway. Taken together, rapamycin may be a promising pharmacological intervention against developmental alterations associated with binge alcohol exposure and may provide future studies with further upstream and downstream pharmacological targets.

## 4 IMPLICATIONS OF mTOR SIGNALING IN ALCOHOL‐EXPOSED PLACENTA

The mTOR signaling pathway is directly regulated in the placenta by sensing oxygen and nutrient availability.^42^ Among nutrients, chronic alcohol use has a targeted negative effect on folate transport through the placenta.^43^ Low gestational folate levels are associated with impaired placental mTOR signaling, which can mediate an inhibition of placental nutrient transporter expression, leading to conditions like intrauterine growth restriction (IUGR).^44^ Similarly, in baboons, fetal weight and mTORC1 signaling were both directly correlated with maternal folate levels.^45^

In humans, the mTORC1 complex is highly expressed in the placental syncytiotrophoblast and decreased mTOR signaling in the placenta was associated with fetal growth restriction.^42^ Interestingly, human placental mTOR signaling was observed to increase in cases of large for gestational age fetuses.^45^ So, the importance of both mTOR potentiation and disruption in the placenta should not be underestimated.

In comparing appropriate for gestational age and IUGR placentas, several downstream signaling proteins of mTORC1 have been analyzed for their phosphorylation states.^45^ 4EBP1 was not differently phosphorylated, while S6K1 was significantly so, indicating that alcohol's influence on intrauterine growth restriction may occur via the S6K1 component of the activated mTORC1 downstream signaling cascade.^45^ Interestingly, in human IUGR cases not limited to alcohol exposure, overall mTOR protein expression in IUGR placentas was found to be 51% higher compared to appropriate for gestational age placentas,^45^ potentially indicating a self‐correcting feedback mechanism to boost activated mTOR signaling.

mTOR is a major signaling component in the cell migration processes of first‐trimester extravillous trophoblasts, a type of differentiated placental cell critical for developing the placental‐uterine interface.^46^ Administration of growth factors which activate mTOR through activation of upstream AKT and PI3K resulted in an increase in the motility of the trophoblast.^46^ Alcohol treatment (20–40 mM for 24 h) on a first‐trimester extravillous trophoblast cell line SGHPL4 was found to reduce proliferation, as well as inhibit amino acid uptake at 40 mM alcohol concentrations.^46^

Later in development, alcohol negatively affects placental uptake of glucose and folic acid, leading to nutrient deficiencies in the placenta and fetus.^46^ Others have noted particular structural deficits. For example, alcohol consumption during pregnancy does not appear to significantly alter placental weight, even as birth weights and placental‐to‐birth weight ratios are significantly affected.^47^ As the placenta is a crucial interface point for the fetus to receive nutrients, altering its mTOR pathway inflicts consequential effects on the developing fetus, even if the placenta itself is only moderately transformed.

## 5 ALTERATIONS IN mTOR SIGNALING IN BONES FOLLOWING ALCOHOL EXPOSURE

Outside of pregnancy, more is known about alcohol's teratogenic effects on mTOR in the bones of adult males and nonpregnant females. Broadly speaking, chronic binge drinking has negative effects on bone remodeling, which can lead to loss of bone mass and eventually osteoporosis.^48^ Alcohol intake in nonpregnant female mice (50 mM for 3 days) induced apoptosis in osteocytes, promoted osteoclast differentiation, suppressed osteogenesis, as well as increased adipogenic differentiation.^49^ In the same study, alcohol exposure led to elevated phosphorylation levels of mTOR and S6K1 in bone marrow mesenchymal stem cells (BMMSCs). Inhibition of the mTOR pathway with rapamycin (1.5 mg/kg/day for 14 days) restored the disruption in BMMSC differentiation induced by heavy alcohol consumption.^49^

In another study, rats were given increasing doses of alcohol in their drinking water (from 5–30% w/v) over a six‐week period to model chronic alcoholism.^50^ Eight out of 10 rats in the alcohol group exhibited severe osteonecrosis of the femoral head. In human bone mesenchymal stem cell culture, alcohol (50 mM) significantly downregulated Osteocalcin (OCN), Collagen 1, and RUNX family transcription factor 2 (RUNX2) expression through activation of the mTOR signaling cascade, as evidenced by significantly increased levels of p‐mTOR and p‐S6K1 (Ser371).^50^ This increased phosphorylation of mTOR was observed in conjunction with an enhanced proliferative capacity of bone mesenchymal stem cells, promoting the development of an age‐related phenotype wherein the bone mesenchymal stem cell's capacity for self‐renewal and its potential for differentiation are reduced. 50 nM rapamycin and 10 mM betaine treatment reversed the inhibition of osteogenic gene expression brought on by alcohol exposure.^50^

Alcohol has also been found to downregulate human dental pulp cell differentiation and mineralization via activation of the mTOR pathway. 50 mM alcohol significantly upregulated the activity of p‐mTOR in dental pulp cells, without altering the viability of those cells.^51^ The upregulation of the mTOR pathway by alcohol in these cells led to a downregulation of known odontoblastic markers as measured by mRNA levels, including OCN, RUNX2, Dentin Sialophosphoprotein, and Dentin Matrix Protein 1. Rapamycin treatment relieved the inhibition of mRNA expression of those markers. This indicates that alcohol may be harmful to the regenerative capabilities of dental pulp cells, and that this phenotype is likely a result of mTOR signaling potentiation.^51^

The growth‐restricted phenotype observed in alcohol‐exposed rat pups is an expected outcome of whole‐body skeletal bone growth restriction. Increased potentiation of mTOR activity appears to be the cause of restricted fetal growth, and further research into this connection is merited.

## 6 ALTERATIONS IN mTOR SIGNALING IN MUSCLE FOLLOWING ALCOHOL EXPOSURE

In adult men and nonpregnant women, skeletal muscle protein synthesis is partially inhibited after acute alcohol intoxication.^52^ Alcohol exposure affects all muscles, but those effects are most noticeable in type II fast‐twitch muscle fibers of chronic alcoholics.^53^ Since alcohol has not been shown to downregulate the total number of ribosomes, an effect on translation has been inferred in these tissues.^52^ It is also speculated that the downstream kinase functionality of mTORC1 activation is suppressed by alcohol exposure, either directly or indirectly, because the total amount of mTOR core and associated proteins was not changed.^52^ Regarding pathway effects, decreases in phosphorylation states in upstream insulin receptor IRS‐1 or protein kinase B are not directly involved in alcohol's effect on mTOR phosphorylation and the alcohol‐induced decrease of phosphorylation of nearby downstream mTOR pathway components involved in translation within skeletal muscle.^53^

Alcohol exposure can also affect mTOR in muscle cells by way of altering how they utilize nutrients. For example, under normal conditions, leucine supplementation enhanced the phosphorylation of eIF4G, S6K1 and mTOR itself, promoting additional protein synthesis. Alcohol treatment appeared to induce a leucine resistance within skeletal muscles, introducing a state where leucine no longer promoted those changes when administered in combination with alcohol.^52^ eIF4E‐BP1, when exposed to alcohol in the gastrocnemius of rats, was found to reduce the phosphorylation of three key threonine residues by 40% to 80%, leading it to return or remain in its inactive eIF4E•eIF4G complex.^52^

Meanwhile, in the heart, an important regulator of the antioxidant response to cardiomyopathy induced by chronic alcohol intake, the protein NRF2, can function as a metabolic switch and regulate downstream targets of mTOR.^54^ Interestingly, in both cardiac and skeletal muscle, alcohol consumption (rats fed 12% to 36% of total caloric intake as alcohol, ad libitum^54^) increases the binding attraction between RAPTOR and DEPTOR, promoting a more closed conformation of the complex, thereby suppressing the activity of mTORC1 and its active downstream components.^55^ The effect of chronic alcohol intake on RAPTOR is a phosphorylation on ser‐792. This residue is naturally phosphorylated by AMPK, and this phosphorylation leads to an inhibition of mTORC1's downstream pathway components.^55^ However, in chronically alcohol‐fed rats, this ser‐792 residue of RAPTOR is phosphorylated without any observable increase in AMPK activity. There is no mechanistic explanation for this effect yet.

Alcohol‐derived pathologies have been extensively studied in adults, but the disruption of fetal muscle development by maternal alcohol consumption is a frontier with great potential for breakthroughs in FASD research. This is especially important area of study, as there are significant metabolic differences between fetal and adult skeletal muscle tissues.

### 6.1 Elevated mTORC2 signaling in myoblasts following alcohol exposure

It is important to note that mTORC2 may also play a role in regulating mTORC1 following alcohol exposure. For instance, alcohol‐exposed C2C12 mouse myoblasts (100 mM, 18–24 hours) revealed elevated levels of total RICTOR, mSin1, PRR5, and DEPTOR, which are all mTORC2 complex components.^56^ RICTOR was then shown to have decreased binding with DEPTOR and 14–3‐3 protein, regulators of metabolic function,^57^ as well as an enhanced association with the remaining aforementioned mTORC2 proteins, demonstrating increased mTORC2 activity.^56^ To further test RICTOR's role in regulating alcohol‐induced changes, cells were transfected with RICTOR shRNA in order to produce RICTOR knockdown cells. Interestingly, RICTOR knockdown cells treated with alcohol revealed that the inhibitory effect on protein synthesis was partially ameliorated, as compared to scrambled shRNA transfected controls.^56^ Noting that mTORC1 activity typically decreases following alcohol exposure,^58^ this may also signal increased mTORC2 activity and a potential positive feedback loop between these complexes. These studies together represent an early step indicating a homeostatic balance between the two mTOR complexes following alcohol treatment. This balance could prove fruitful in the search for more mechanistic causes within the field of fetal development and maternal alcohol consumption.

## 7 CONCLUSION

In this review we have aggregated supported works that depict mTOR as a clear linchpin in alcohol‐impaired fetal development and non‐pregnant adult binge‐alcohol paradigms. Recent studies have elucidated the structure of this signaling pathway, allowing us to mechanistically peer into how the mTOR signaling cascade transduces cues from binge alcohol exposure into molecular action. mTORC1 in particular contributes to many processes implicated in alcohol pathology, including protein synthesis, autophagy, nutrient transport, and cell proliferation, however little is known about mTORC2 and its role in FASD. We have also considered mTOR as a potential target in various tissues for future FASD studies and have noted mTOR as a possible target for pharmacological intervention.

## AUTHOR CONTRIBUTIONS

**Alexander L. Carabulea:** Writing—Original Draft, Conceptualization, Writing—Review & Editing, Draft manuscript preparation; **Joseph D. Janeski:** Writing—Original Draft, Writing—Review & Editing; **Vishal D. Naik:** Supervision, Visualization, Writing—Review & Editing. **Kang Chen:** Review & Editing; **Gil Mor:** Review & Editing; **Jayanth Ramadoss:** Funding acquisition, Supervision, Conceptualization, Writing—Review & Editing.

## FUNDING INFORMATION

This study was supported by National Institutes of Health [HL151497 (JR), AA23520 (JR), AA23035 (JR)].

## DISCLOSURES

The authors declare no conflicts of interest.

## Data Availability

Data sharing not applicable to this article as no datasets were generated or analyzed during the current study.

## References

[1] Sokol RJ , Delaney‐Black V , Nordstrom B . Fetal alcohol spectrum disorder. JAMA. 2003;290:2996‐2999. 14665662 10.1001/jama.290.22.2996

[2] Jones KL , Smith DW . The fetal alcohol syndrome. Teratology. 1975;12:1‐10. 1162620 10.1002/tera.1420120102

[3] Carmona RH . Advisory on Alcohol Use in Pregnancy. (Centers for Disease Control and Prevention , ed.). U.S. Surgeon General; 2005.

[4] Williams JF , Smith VC , Committee On Substance Abuse . Fetal alcohol Spectrum disorders. Pediatrics. 2015;136:e1395‐e1406. 26482673 10.1542/peds.2015-3113

[5] Angira D , Shaik A , Thiruvenkatam V . Structural and strategic landscape of PIKK protein family and their inhibitors: an overview. Front Biosci (Landmark Ed). 2020;25:1538‐1567. 32114444 10.2741/4867

[6] Liu GY , Sabatini DM . mTOR at the nexus of nutrition, growth, ageing and disease. Nat Rev Mol Cell Biol. 2020;21:183‐203. 31937935 10.1038/s41580-019-0199-y PMC7102936

[7] Lovejoy CA , Cortez D . Common mechanisms of PIKK regulation. DNA Repair (Amst). 2009;8:1004‐1008. 19464237 10.1016/j.dnarep.2009.04.006 PMC2725225

[8] Zarogoulidis P , Lampaki S , Turner JF , et al. mTOR pathway: a current, up‐to‐date mini‐review (Review). Oncol Lett. 2014;8:2367‐2370. 25360163 10.3892/ol.2014.2608 PMC4214394

[9] Yang H , Rudge DG , Koos JD , Vaidialingam B , Yang HJ , Pavletich NP . mTOR kinase structure, mechanism and regulation. Nature. 2013;497:217‐223. 23636326 10.1038/nature12122 PMC4512754

[10] Saxton RA , Sabatini DM . mTOR signaling in growth, metabolism, and disease. Cell. 2017;168:960‐976. 28283069 10.1016/j.cell.2017.02.004 PMC5394987

[11] Tan VP , Miyamoto S . Nutrient‐sensing mTORC1: integration of metabolic and autophagic signals. J Mol Cell Cardiol. 2016;95:31‐41. 26773603 10.1016/j.yjmcc.2016.01.005 PMC4909545

[12] Szwed A , Kim E , Jacinto E . Regulation and metabolic functions of mTORC1 and mTORC2. Physiol Rev. 2021;101:1371‐1426. 33599151 10.1152/physrev.00026.2020 PMC8424549

[13] Pullen N , Dennis PB , Andjelkovic M , et al. Phosphorylation and activation of p70s6k by PDK1. Science. 1998;279:707‐710. 9445476 10.1126/science.279.5351.707

[14] Burnett PE , Barrow RK , Cohen NA , Snyder SH , Sabatini DM . RAFT1 phosphorylation of the translational regulators p70 S6 kinase and 4E‐BP1. Proc Natl Acad Sci U S A. 1998;95:1432‐1437. 9465032 10.1073/pnas.95.4.1432 PMC19032

[15] Hannan KM , Brandenburger Y , Jenkins A , et al. mTOR‐dependent regulation of ribosomal gene transcription requires S6K1 and is mediated by phosphorylation of the carboxy‐terminal activation domain of the nucleolar transcription factor UBF. Mol Cell Biol. 2003;23:8862‐8877. 14612424 10.1128/MCB.23.23.8862-8877.2003 PMC262650

[16] Mayer C , Zhao J , Yuan X , Grummt I . mTOR‐dependent activation of the transcription factor TIF‐IA links rRNA synthesis to nutrient availability. Genes Dev. 2004;18:423‐434. 15004009 10.1101/gad.285504 PMC359396

[17] Michels AA , Robitaille AM , Buczynski‐Ruchonnet D , et al. mTORC1 directly phosphorylates and regulates human MAF1. Mol Cell Biol. 2010;30:3749‐3757. 20516213 10.1128/MCB.00319-10 PMC2916396

[18] Shor B , Wu J , Shakey Q , et al. Requirement of the mTOR kinase for the regulation of Maf1 phosphorylation and control of RNA polymerase III‐dependent transcription in cancer cells. J Biol Chem. 2010;285:15380‐15392. 20233713 10.1074/jbc.M109.071639 PMC2865278

[19] Holz MK , Ballif BA , Gygi SP , Blenis J . mTOR and S6K1 mediate assembly of the translation preinitiation complex through dynamic protein interchange and ordered phosphorylation events. Cell. 2005;123:569‐580. 16286006 10.1016/j.cell.2005.10.024

[20] Dorrello NV , Peschiaroli A , Guardavaccaro D , Colburn NH , Sherman NE , Pagano M . S6K1‐ and betaTRCP‐mediated degradation of PDCD4 promotes protein translation and cell growth. Science. 2006;314:467‐471. 17053147 10.1126/science.1130276

[21] Ma XM , Yoon SO , Richardson CJ , Julich K , Blenis J . SKAR links pre‐mRNA splicing to mTOR/S6K1‐mediated enhanced translation efficiency of spliced mRNAs. Cell. 2008;133:303‐313. 18423201 10.1016/j.cell.2008.02.031

[22] Kim J , Kundu M , Viollet B , Guan KL . AMPK and mTOR regulate autophagy through direct phosphorylation of Ulk1. Nat Cell Biol. 2011;13:132‐141. 21258367 10.1038/ncb2152 PMC3987946

[23] Hosokawa N , Hara T , Kaizuka T , et al. Nutrient‐dependent mTORC1 association with the ULK1‐Atg13‐FIP200 complex required for autophagy. Mol Biol Cell. 2009;20:1981‐1991. 19211835 10.1091/mbc.E08-12-1248 PMC2663915

[24] Ganley IG , Lam du H , Wang J , Ding X , Chen S , Jiang X . ULK1.ATG13.FIP200 complex mediates mTOR signaling and is essential for autophagy. J Biol Chem. 2009;284:12297‐12305. 19258318 10.1074/jbc.M900573200 PMC2673298

[25] Dikic I , Elazar Z . Mechanism and medical implications of mammalian autophagy. Nat Rev Mol Cell Biol. 2018;19:349‐364. 29618831 10.1038/s41580-018-0003-4

[26] Settembre C , Zoncu R , Medina DL , et al. A lysosome‐to‐nucleus signalling mechanism senses and regulates the lysosome via mTOR and TFEB. EMBO J. 2012;31:1095‐1108. 22343943 10.1038/emboj.2012.32 PMC3298007

[27] Roczniak‐Ferguson A , Petit CS , Froehlich F , et al. The transcription factor TFEB links mTORC1 signaling to transcriptional control of lysosome homeostasis. Sci Signal. 2012;5:ra42. 22692423 10.1126/scisignal.2002790 PMC3437338

[28] Kim W‐Y . Brain size is controlled by the mammalian target of rapamycin (mTOR) in mice. Commun Integr Biol. 2015;8:e994377. 26845545 10.4161/19420889.2014.994377 PMC4594332

[29] Gutala R , Wang J , Kadapakkam S , Hwang Y , Ticku M , Li MD . Microarray analysis of ethanol‐treated cortical neurons reveals disruption of genes related to the ubiquitin‐proteasome pathway and protein synthesis. Alcohol Clin Exp Res. 2004;28:1779‐1788. 15608593 10.1097/01.alc.0000148117.17707.b4

[30] Davis‐Anderson KL , Wesseling H , Siebert LM , et al. Fetal regional brain protein signature in FASD rat model. Reprod Toxicol. 2018;76:84‐92. 29408587 10.1016/j.reprotox.2018.01.004 PMC5834402

[31] Lee J , Lunde‐Young R , Naik V , Ramirez J , Orzabal M , Ramadoss J . Chronic binge alcohol exposure during pregnancy alters mTOR system in rat fetal hippocampus. Alcohol Clin Exp Res. 2020;44:1329‐1336. 32333810 10.1111/acer.14348 PMC7328280

[32] Lopatynska‐Mazurek M , Pankowska A , Gibula‐Tarlowska E , Pietura R , Kotlinska JH . Rapamycin improves recognition memory and normalizes amino‐acids and amines levels in the hippocampal dentate gyrus in adult rats exposed to ethanol during the neonatal period. Biomolecules. 2021;11:362. 33673489 10.3390/biom11030362 PMC7997340

[33] Chang RC , Thomas KN , Mehta NA , Veazey KJ , Parnell SE , Golding MC . Programmed suppression of oxidative phosphorylation and mitochondrial function by gestational alcohol exposure correlate with widespread increases in H3K9me2 that do not suppress transcription. Epigenetics Chromatin. 2021;14:27. 34130715 10.1186/s13072-021-00403-w PMC8207718

[34] Chen G , Ke Z , Xu M , et al. Autophagy is a protective response to ethanol neurotoxicity. Autophagy. 2012;8:1577‐1589. 22874567 10.4161/auto.21376 PMC3494588

[35] Tanida I , Ueno T , Kominami E . LC3 and autophagy. Methods Mol Biol. 2008;445:77‐88. 18425443 10.1007/978-1-59745-157-4_4

[36] Pascual M , Lopez‐Hidalgo R , Montagud‐Romero S , Urena‐Peralta JR , Rodriguez‐Arias M , Guerri C . Role of mTOR‐regulated autophagy in spine pruning defects and memory impairments induced by binge‐like ethanol treatment in adolescent mice. Brain Pathol. 2021;31:174‐188. 32876364 10.1111/bpa.12896 PMC8018167

[37] Girault V , Gilard V , Marguet F , et al. Prenatal alcohol exposure impairs autophagy in neonatal brain cortical microvessels. Cell Death Dis. 2017;8:e2610. 28182007 10.1038/cddis.2017.29 PMC5386476

[38] Costa‐Mattioli M , Monteggia LM . mTOR complexes in neurodevelopmental and neuropsychiatric disorders. Nat Neurosci. 2013;16:1537‐1543. 24165680 10.1038/nn.3546

[39] Bockaert J , Marin P . mTOR in brain physiology and pathologies. Physiol Rev. 2015;95:1157‐1187. 26269525 10.1152/physrev.00038.2014

[40] Lopatynska‐Mazurek M , Antolak A , Grochecki P , et al. Rapamycin improves spatial learning deficits, vulnerability to alcohol addiction and altered expression of the GluN2B subunit of the NMDA receptor in adult rats exposed to ethanol during the neonatal period. Biomolecules. 2021;11:650. 33924998 10.3390/biom11050650 PMC8147055

[41] Luo J . Autophagy and ethanol neurotoxicity. Autophagy. 2014;10:2099‐2108. 25484085 10.4161/15548627.2014.981916 PMC4502825

[42] Hart B , Morgan E , Alejandro EU . Nutrient sensor signaling pathways and cellular stress in fetal growth restriction. J Mol Endocrinol. 2019;62:R155‐R165. 30400060 10.1530/JME-18-0059 PMC6443503

[43] Hutson JR , Stade B , Lehotay DC , Collier CP , Kapur BM . Folic acid transport to the human fetus is decreased in pregnancies with chronic alcohol exposure. PLoS ONE. 2012;7:e38057. 22666445 10.1371/journal.pone.0038057 PMC3362577

[44] Rosario FJ , Nathanielsz PW , Powell TL , Jansson T . Maternal folate deficiency causes inhibition of mTOR signaling, down‐regulation of placental amino acid transporters and fetal growth restriction in mice. Sci Rep. 2017;7:3982. 28638048 10.1038/s41598-017-03888-2 PMC5479823

[45] Dong J , Shin N , Chen SQ , Lei J , Burd I , Wang XH . Is there a definite relationship between placental mTOR signaling and fetal growth? Biol Reprod. 2020;103:471‐486. 32401303 10.1093/biolre/ioaa070

[46] Correia‐Branco A , Keating E , Martel F . Involvement of mTOR, JNK and PI3K in the negative effect of ethanol and metformin on the human first‐trimester extravillous trophoblast HTR‐8/SVneo cell line. Eur J Pharmacol. 2018;833:16‐24. 29807029 10.1016/j.ejphar.2018.05.038

[47] Wang N , Tikellis G , Sun C , et al. The effect of maternal prenatal smoking and alcohol consumption on the placenta‐to‐birth weight ratio. Placenta. 2014;35:437‐441. 24816479 10.1016/j.placenta.2014.04.006 PMC4096564

[48] Cheraghi Z , Doosti‐Irani A , Almasi‐Hashiani A , et al. The effect of alcohol on osteoporosis: a systematic review and meta‐analysis. Drug Alcohol Depend. 2019;197:197‐202. 30844616 10.1016/j.drugalcdep.2019.01.025

[49] Liu Y , Kou X , Chen C , et al. Chronic high dose alcohol induces osteopenia via activation of mTOR signaling in bone marrow mesenchymal stem cells. Stem Cells. 2016;34:2157‐2168. 27145264 10.1002/stem.2392

[50] Yang Q , Yin W , Chen Y , et al. Betaine alleviates alcohol‐induced osteonecrosis of the femoral head via mTOR signaling pathway regulation. Biomed Pharmacother. 2019;120:109486. 31586901 10.1016/j.biopha.2019.109486

[51] Qin W , Huang QT , Weir MD , et al. Alcohol inhibits odontogenic differentiation of human dental pulp cells by activating mTOR signaling. Stem Cells Int. 2017;2017:8717454. 29062364 10.1155/2017/8717454 PMC5618757

[52] Lang CH , Frost RA , Deshpande N , et al. Alcohol impairs leucine‐mediated phosphorylation of 4E‐BP1, S6K1, eIF4G, and mTOR in skeletal muscle. Am J Physiol Endocrinol Metab. 2003;285:E1205‐E1215. 12944322 10.1152/ajpendo.00177.2003

[53] Lang CH , Frost RA , Summer AD , Vary TC . Molecular mechanisms responsible for alcohol‐induced myopathy in skeletal muscle and heart. Int J Biochem Cell Biol. 2005;37:2180‐2195. 15982919 10.1016/j.biocel.2005.04.013

[54] Korzick DH , Sharda DR , Pruznak AM , Lang CH . Aging accentuates alcohol‐induced decrease in protein synthesis in gastrocnemius. Am J Physiol Regul Integr Comp Physiol. 2013;304:R887‐R898. 23535459 10.1152/ajpregu.00083.2013 PMC3652166

[55] Kimball SR , Lang CH . Mechanisms underlying muscle protein imbalance induced by alcohol. Annu Rev Nutr. 2018;38:197‐217. 30130465 10.1146/annurev-nutr-071816-064642 PMC6377942

[56] Hong‐Brown LQ , Brown CR , Navaratnarajah M , Huber DS , Lang CH . Alcohol‐induced modulation of rictor and mTORC2 activity in C2C12 myoblasts. Alcohol Clin Exp Res. 2011;35:1445‐1453. 21438886 10.1111/j.1530-0277.2011.01480.x PMC3503252

[57] Kleppe R , Martinez A , Doskeland SO , Haavik J . The 14‐3‐3 proteins in regulation of cellular metabolism. Semin Cell Dev Biol. 2011;22:713‐719. 21888985 10.1016/j.semcdb.2011.08.008

[58] Hong‐Brown LQ , Brown CR , Huber DS , Lang CH . Alcohol and indinavir adversely affect protein synthesis and phosphorylation of MAPK and mTOR signaling pathways in C2C12 myocytes. Alcohol Clin Exp Res. 2006;30:1297‐1307. 16899032 10.1111/j.1530-0277.2006.00157.x

